# Editorial: Mitochondrial Dysfunction and Neurodegeneration

**DOI:** 10.3389/fnins.2019.01372

**Published:** 2019-12-18

**Authors:** Victor Tapias

**Affiliations:** Feil Family Brain and Mind Research Institute, Weill Cornell Medicine, New York, NY, United States

**Keywords:** mitochondrial dysfunction, quality control, neurodegenerative diseases, Parkinson's disease, Alzheimer's disease

Neurodegenerative diseases are incurable and inexorably progressive conditions that affect the central nervous system and result in a selective pattern of neuronal death. Parkinson's disease (PD) and Alzheimer's disease (AD) are the most common neurodegenerative diseases. While most cases are idiopathic, studies have confirmed that genetic factors contribute to the pathogenesis of both PD and AD. PD is characterized by loss of dopamine (DA)-producing neurons of the substantia nigra, and as a consequence of a reduction in striatal DA content. A neuropathological hallmark is the presence of Lewy body inclusions in many of the remaining neurons and Lewy-neurite pathology in the neuropil. The classic histopathological hallmarks of AD are the extracellular accumulation of amyloid-β (Aβ) plaques and intracellular deposition of hyperphosphorylated tau into neurofibrillary tangles. Despite distinct clinical and pathological features, the formation of misfolded protein aggregates is a common feature of neurodegenerative diseases, which can be mainly classified into synucleinopathies, tauopathies, and amyloidopathies. Neurodegenerative diseases share critical processes, such as mitochondrial anomalies, oxidative damage, and inflammation that are implicated in the gradual loss of neuronal function and cell death.

A plethora of reports indicate that mitochondrial dysfunction is a central factor in the pathophysiology of neurodegenerative diseases (Lin and Beal, 2006; Tapias et al., 2017, 2018, 2019). Elevated oxidative stress can damage the mitochondrial respiratory chain. Mitochondrial complexes I and III and the mitochondrially located monoamine oxidase (MAO) B are the main source of reactive oxygen and nitrogen species. A region-dependent regulation of MAO has been reported in PD and AD (Tong et al., 2017; Quartey et al.). Furthermore, perturbations in mitochondrial dynamics, mitochondrial transport within axons, mitophagy, and accumulation of somatic mtDNA mutations are associated with impaired mitochondrial function. Compromised mitochondrial quality control mechanisms may lead to the accumulation of defective mitochondria and concomitant oxidative damage, defective calcium (Ca^2+^) homeostasis and signaling, synaptic pathology, and ferroptotic neuronal death. The present Research Topic aims to critically evaluate the current literature on molecular mechanisms associated with neurodegenerative diseases and it provides novel insights into disturbances in mitochondrial function, which occur during neurodegeneration. This topic also suggests that the development of novel mitochondria-targeted therapeutic strategies may be useful in the treatment of neurodegenerative diseases.

Mechanisms for the maintenance of mitochondrial integrity and functionality are crucial for neuronal survival. Mitochondrial dynamics play a key role in ensuring mitochondrial quality control and are tightly regulated by the fusion/fission machinery, which allows the formation or degradation of a mitochondrial syncytium. The molecular process of fusion is driven by the GTPases Opa1 and Mitofusin-1 (Mfn1) and Mfn2 while dynamin-related protein (Drp1) interacts with the mitochondrial fission 1 protein (Fis1), mitochondrial fission factor (Mff) and mitochondrial dynamics proteins of 49 and 51 kDa (MiD49/51) to mediate mitochondrial fission.It has been recently shown that mitofilin (Mic60), a component of the MICOS complex that plays a key role in the maintenance of mitochondrial structure and function,can regulate mitochondrial dynamics (Li et al., 2016; Van Laar et al., 2016; Van Laar et al.). Axonal transport, a cellular mechanism responsible for the active trafficking of lipids, proteins, neurotransmitters, and organelles, is essential for neuronal network function and viability. Anterograde transport carries new synthesized material from the cell body to distal axons and is mediated by kinesin motor proteins. Dynein-driven retrograde transport is required for efficient distribution of cargoes from the axon terminals toward the soma. Mitochondrial movement along both microtubule and actin filaments is regulated by a motor adaptor complex that attaches the anterograde kinesin-1 motor and retrograde dynein motor to the outer mitochondrial membrane, in a process mediated by the membrane-anchored Miro (RhoT1/2) and Milton (Trak1/2) proteins (Schwarz, 2013). Decreased mitochondrial trafficking within axons accompanied by inhibited neurite outgrowth was found in cultures of dorsal root ganglia sensory neurons overexpressing the muscarinic acetylcholine type 1 receptor (Sabbir et al.). There is growing evidence of a crosstalk between fusion-fission and axonal flux mitochondrial dynamics and axonal transport integrity (Misgeld and Schwarz, 2017; Tapias et al., 2017; Franco-Iborra et al.; Perez et al.).

Mitophagy is a specialized type of autophagy that mediates the clearance of damaged mitochondria by lysosomes. Mitochondrial autophagy is inextricably linked to protein import since the translocation of the PTEN-induced putative kinase 1 (Pink1) into the mitochondrial inner membrane via the Tim/Tom complex plays a pivotal role in regulating Pink1/Parkin-mediated mitophagy (Poole et al., 2008; Geisler et al., 2010; Vives-Bauza et al., 2010). Moreover, impaired lysosomal degradation can impact mitochondria by causing mitophagy deficits; aminochrome, a product of DA oxidation and the precursor of neuromelanin, induces mitochondrial dysfunction by blocking the selective clearance of damaged mitochondria by autophagy (Segura-Aguilar and Huenchuguala). Protein post-translational modifications such as enzymatic glycosylation and non-enzymatic glycation together with a disruption of the mitochondrial quality control system, result in defective mitophagy and excessive accumulation of dysfunctional proteins (Videira and Castro-Caldas). Altered autophagy phenotypes have recently been associated with optineurin, a multifunctional cargo adaptor protein observed in diverse brain regions of rats after exposure to rotenone (Wise et al.). Mitochondria contribute to aging, mitochondrial-related diseases, and neurodegeneration through the accumulation of somatic mtDNA mutations—point mutations and large-scale deletions (Simon et al., 2001; Dolle et al., 2016; Hoekstra et al., 2016; Chinnery and Gomez-Duran; Emperador et al.). Point mutations are likely to arise from an inefficient base excision repair system while mtDNA deletions and rearrangements may result from errors in replication and/or double-strand break repair (Krishnan et al., 2008). Although the precise mechanism by which mtDNA damage contributes to both aging phenotypes and neurodegeneration remains unclear, a direct relationship between age-related oxidative damage to mtDNA and oxidation of glutathione has been reported in the brains of mice and rats (de la Asuncion et al., 1996).

It has also been reported that there is a link between impaired mitochondrial function and depression (Bansal Kuhad and Kuhad, 2016; Allen et al.). Patients suffering from depression show reduced glucose metabolism in different regions of the brain (Baxter et al., 1989; Gardner et al., 2003). Hypothalamic-pituitary-adrenal axis hyperactivity has been implicated in the upregulation of glucocorticoid synthesis in depression, which plays a pivotal biphasic role in modulating mitochondrial functions. Indeed, following acute and chronic immobilization-induced stress, glucocorticoid receptors regulated the expression of several mitochondrial genes in the rat hippocampus (Hunter et al., 2016).

Sustained synaptic release of glutamate, the primary excitatory neurotransmitter in the mammalian central nervous system and the metabolic precursor for the inhibitory neurotransmitter gamma-aminobutyric acid (GABA), results in the overactivation of the N-methyl-D-aspartate (NMDA) receptors and the subsequent loss of ionic homeostasis and excessive influx of Ca^2+^ into the cell, which causes excitotoxicity. Ca^2+^ is the most important signaling entity in neurons and its levels are tightly regulated by organelles such as mitochondria and the ER and by buffering through Ca^2+^-binding proteins, such as calmodulin, calbindin, and parvalbumin. As shown in some manuscripts of this Research Topic, disruption of the processes underlying Ca^2+^ homeostasis and signaling have been consistently observed in neurodegenerative diseases and glaucoma (Cheung et al.; Muller et al.; Verma et al.; Barodia et al., 2019; Schrank et al., 2019). The acidic C-terminus of α-synuclein (α-syn) contains a Ca^2+^-binding domain and a transient increase in free intracellular Ca^2+^ can accelerate α-syn aggregation (Nath et al., 2011; Follett et al., 2013). Oligomeric forms of α-syn can exacerbate the intracellular concentration of Ca^2+^ by forming pore-like structures in the plasma membrane (Pacheco et al., 2015). α-Syn can interact with calmodulin in a Ca^2+^-dependent manner, resulting in an increased rate of α-syn fibrillation (Martinez et al., 2003). α-Syn causes sustained elevations of cytosolic Ca^2+^ and it initiates a toxic calmodulin–calcineurin cascade, which contributes to DA neuronal death (Caraveo et al., 2014; Luo et al., 2014). Disturbances in Ca^2+^ homeostasis promote Aβ formation and tau hyperphosphorylation (Buxbaum et al., 1994; LaFerla, 2002; Mattson and Chan, 2003). There are deleterious effects of presenilin 1 and synthetic Aβ oligomers in producing Ca^2+^ dysregulation, which can induce a rapid Ca^2+^ release mediated by the ryanodine and inositol triphosphate receptors (Mattson et al., 1992; Stutzmann et al., 2003; Demuro et al., 2005). *In vivo* experiments have shown that Aβ plaque deposition promotes Ca^2+^ overload and calcineurin activation, which leads to downstream synaptic and dendritic spine pathology (Kuchibhotla et al., 2008; Wu et al., 2010). Age-dependent alterations in mitochondrial Ca^2+^ efflux accelerate memory deficits and increase both amyloidosis and tau hyperphosphorylation in 3xTg-AD mice (Jadiya et al., 2019). Rescue of the expression of NCLX (a critical component of the mitochondrial Na^+^/Ca^2+^ exchange) in these mice restored cognitive function and attenuated hippocampal neuronal degeneration. Elevated Ca^2+^ influx plays a key role in promoting pathological tau phosphorylation via modulation of Ca^2+^-binding proteins and/or dysregulation of the enzymatic activity of kinases and phosphatases (Zempel et al., 2010; Mairet-Coello et al., 2013).

Two main pathways cell death have been distinguished, namely apoptosis (programmed cell death) and necrosis (accidental cell death). Ferroptosis, a term coined in 2012, is a form of regulated cell death induced by erastin which is characterized by the iron-dependent accumulation of lipid hydroperoxides with a genetic, morphological, and biochemical profile different from apoptosis and necrosis (Dixon et al., 2012). Several biological processes determine the sensitivity to ferroptosis, such as the metabolism of amino acids, polyunsaturated fatty acids, and iron as well as the biosynthesis of glutathione, NADPH, coenzyme Q10, selenium, and phospholipids (Stockwell et al., 2017). Evidence supporting an involvement of ferroptosis in the pathogenesis of neurodegenerative diseases include iron accumulation, lipid peroxidation, depletion of GSH, and mutations in the transferrin and cerulopasmin encoding gene (Guiney et al., 2017). Deficient regulation of ferroptosis has been described in PD. Toxin-mediated ferroptotic activation was observed in LUHMES cells, MPTP-treated mice, and organotypic slice cultures (Do Van et al., 2016). The conversion of arachidonic acid—one of the main substrates of lipid peroxidation for ferroptosis—to polar degradation products was substantially accelerated in the hippocampus of different transgenic mouse models of AD as well as in post-mortem hippocampal tissue form patients with AD (Furman et al., 2016). Ferroptotic cell death can be triggered through diverse mechanisms. Upregulation of the selenoenzyme glutathione peroxidase 4 activity or treatment with ferroptosis inhibitors can confer neuroprotection in different cellular and animal models of PD and AD (Friedmann Angeli et al., 2014; Do Van et al., 2016; Guiney et al., 2017; Hambright et al., 2017). The nuclear factor erythroid-2-related factor 2 (Nrf2) transcriptionally regulates numerous genes involved in both oxidative damage and inflammation, which are implicated in ferroptosis. It indirectly controls the lipid content that is a critical determinant of sensitivity to ferroptotic cell death (Doll et al., 2017). Therefore, it has been suggested that compounds which target Nrf2 may counteract ferroptotic-mediated neuronal loss and exert beneficial effects in the treatment of neurogenerative diseases (Abdalkader et al.). Although pathologically-related aggregate species of α-syn, Aβ and tau regulate lipid peroxidation, glutathione levels, and iron homeostasis, as yet no studies have explored their potential role in ferroptotic cell death.

In conclusion, this special issue provides scientists and clinicians with new insights into the molecular mechanisms underlying the role of mitochondrial dysfunction in the pathophysiology of neurodegenerative diseases such as PD and AD. Furthermore, it may provide further rationale for the development of effective therapeutic interventions targeting mitochondria to treat these devastating illnesses.

## Author Contributions

VT confirms being the sole contributor of this work and has approved it for publication.

### Conflict of Interest

The author declares that the research was conducted in the absence of any commercial or financial relationships that could be construed as a potential conflict of interest.
